# Liraglutide, a glucagon-like peptide-1 receptor agonist, inhibits bone loss in an animal model of osteoporosis with or without diabetes

**DOI:** 10.3389/fendo.2024.1378291

**Published:** 2024-05-29

**Authors:** Zongyi Wu, Wei Deng, Yiming Ye, Jie Xu, Deyu Han, Yu Zheng, Qun Zheng

**Affiliations:** ^1^ Department of Orthopedics, The Second Affiliated Hospital and Yuying Children’s Hospital of Wenzhou Medical University, Wenzhou, China; ^2^ Department of Rheumatology Immunology, The Second Affiliated Hospital and Yuying Children’s Hospital of Wenzhou Medical University, Wenzhou, China; ^3^ Department of Nephrology, The Second Affiliated Hospital and Yuying Children’s Hospital of Wenzhou Medical University, Wenzhou, China

**Keywords:** liraglutide, osteoporosis, diabetes, efficacy, possible mechanisms

## Abstract

**Introduction:**

Liraglutide (Lrg), a novel anti-diabetic drug that mimics the endogenous glucagon-like peptide-1 to potentiate insulin secretion, is observed to be capable of partially reversing osteopenia. The aim of the present study is to further investigate the efficacy and potential anti-osteoporosis mechanisms of Lrg for improving bone pathology, bone- related parameters under imageology, and serum bone metabolism indexes in an animal model of osteoporosis with or without diabetes.

**Methods:**

Eight databases were searched from their inception dates to April 27, 2024. The risk of bias and data on outcome measures were analyzed by the CAMARADES 10-item checklist and Rev-Man 5.3 software separately.

**Results:**

Seventeen eligible studies were ultimately included in this review. The number of criteria met in each study varied from 4/10 to 8/10 with an average of 5.47. The aspects of blinded induction of the model, blinding assessment of outcome and sample size calculation need to be strengthened with emphasis. The pre-clinical evidence reveals that Lrg is capable of partially improving bone related parameters under imageology, bone pathology, and bone maximum load, increasing serum osteocalcin, N-terminal propeptide of type I procollagen, and reducing serum c-terminal cross-linked telopeptide of type I collagen (P<0.05). Lrg reverses osteopenia likely by activating osteoblast proliferation through promoting the Wnt signal pathway, p-AMPK/PGC1α signal pathway, and inhibiting the activation of osteoclasts by inhibiting the OPG/RANKL/RANK signal pathway through anti-inflammatory, antioxidant and anti-autophagic pathways. Furthermore, the present study recommends that more reasonable usage methods of streptozotocin, including dosage and injection methods, as well as other types of osteoporosis models, be attempted in future studies.

**Discussion:**

Based on the results, this finding may help to improve the priority of Lrg in the treatment of diabetes patients with osteoporosis.

## 1 Introduction

The World Health Organization (WHO) defined osteoporosis as a progressive systemic skeletal disease characterized by low bone mass and microarchitectural deterioration of bone tissue, leading to increased bone fragility and susceptibility to fracture (1, 2). Aside from established risk factors including age, cigarette smoking, low physical activity, the use of drugs such as glucocorticoids, and low calcium and vitamin D levels (3, 4), diabetes has recently gained increased attention as a potential risk factor for osteoporosis and fragility fractures (5, 6). The likely reasons are related to insulin deficiency (7) and the impact of high glucose on calcium and phosphorus metabolism (8). Given that diabetes is a systemic disease associated with a range of chronic and severe complications, the disability and mortality rates are high once fractures occur in patients (6). Although conventional anti-osteoporosis drugs such as calcium tablets, vitamin D, bisphosphonates, denosumab, and teriparatide have been used to treat osteoporosis (2), they do not address the sustained effects of insulin deficiency and high glucose toxicity on bone metabolism. Therefore, besides conventional treatments, it is advantageous to explore drugs that offer both hypoglycemic and anti-osteoporosis effects.

New therapies for diabetes such as glucagon-like peptide-1 receptor agonists (GLP1Ras) have been shown to exert multiple effects on various organs and tissues, including the cardiovascular system (9–12), arteries (13–15), lipid metabolism (16), and bone metabolism (17, 18). Liraglutide (Lrg), a representative GLP1Ras, is a novel anti-diabetic and widely used drug that mimics the endogenous GLP-1 to potentiate insulin secretion (19). Studies have demonstrated that osteoblastic cells express functional receptors for GLP-1 (20), and continuous subcutaneous infusion of GLP-1 or Lrg in diabetes-related osteoporosis models normalized their impaired trabecular architecture and promoted bone formation (8, 21). These findings highlight the potential use of Lrg in combating diabetes-related bone loss. However, the evidence provided by a single literature source is limited, and the mechanism of Lrg for osteoporosis- or diabetes-related osteoporosis has not been systematically summarized. Thus, the present study aims to investigate the pre-clinical evidence and possible mechanisms of Lrg in animal models of osteoporosis.

## 2 Methods

The Preferred Reporting Items for Systematic Review and Meta Analyses (PRISMA) checklist was used to structure this study (22).

### 2.1 Data sources and search strategies

A literature search was conducted to identify all published animal experimental studies of Lrg for osteoporosis in PubMed, EMBASE, Cochrane library, Web of Science database, WanFang, Chinese Science and Technology Journal Database, Chinese Biomedical Database, and China National Knowledge Infrastructure from their inception dates to April 27, 2024. “Liraglutide OR Victoza” AND “Osteoporosis OR Bone Loss OR Osteopenia OR Bone Metabolism” were used as the search terms in PubMed and were modified to suit other databases. A complete record of search strings in PubMed is provided as an example in **Appendix 1**. Additionally, the reference lists of potential articles were searched for relevant studies.

### 2.2 Eligibility criteria

The studies were screened by two independent authors (ZW and WD) and included if they met the following criteria: (1) studies assessing the efficacy of Lrg for osteoporosis or bone loss in animal models were included, (2) the treatment group used Lrg as monotherapy with unrestricted medicament type, dosage, duration, and route of administration, compared with a blank control or placebo in the control group, and (4) bone pathology and/or bone mineral density [including lumbar spine bone mineral density (L-BMD) and femur bone mineral density (F-BMD)] and/or bone histomorphometric parameters under micro-CT [trabecular number (Tb.N) and trabecular thickness (Tb.Th)] and/or bone maximum load and/or bone turnover markers [C-terminal cross-linked telopeptide of type I collagen (CTX), N-terminal propeptide of type I procollagen (PINP), and osteocalcin (OC)] and/or indicators of adverse reactions were selected as the primary outcome measures. Indicators reflecting the mechanisms of anti-osteoporosis action of Lrg were selected as secondary outcome measures. Studies were excluded if they (1) were not controlled experiments or *in vivo* animal experiments, (2) included combination medication in the treatment group, (3) lacked primary outcome indicators or had incomplete data, (4) had inconsistencies between graphic and textual data, and (5) were duplicate publications.

### 2.3 Data extraction

Two reviewers (ZW and YY) independently and systematically performed data extraction, focusing on study design characteristics, animal information, modeling methods, anesthetic details, interventions, and outcomes. Only data pertaining to the highest dose and the final time point were included when the experiments featured multiple Lrg dose groups or various measurement times. Graphical data were measured using Photoshop when results were only available in graphic from and no response was received from the corresponding authors.

### 2.4 Risk of bias in individual studies

Two independent authors (WD and JX) utilized the CAMARADES 10-item quality checklist (23) with minor modifications to assess study quality. The modifications included F—anesthetics without significant bone toxicity or protective activity and G—appropriate animal model with complications or risk factors (including aged, diabetes, hyperlipemia, or hypertensive). The authors first independently selected studies, extracted data, and scored the studies and then discussed disagreements with the corresponding author (QZ) until a consensus was reached.

### 2.5 Statistical analysis

We performed all of the analyses available using RevMan 5.3 software. For continuous data, standardized mean differences (SMDs) and 95% confidence intervals (95% CIs) were calculated to estimate the combined overall effect sizes. Heterogeneity was assessed using the Cochrane Q-statistic test (*P* < 0.05 was considered statistically significant) and the *I* ^2^ statistic test (*I* ^2^ < 50% was considered homogeneous). Data were aggregated using a random-effects model if there was high heterogeneity (*I* ^2^ > 50%); otherwise, a fixed-effects model was adopted. Potential publication bias was assessed by a visual inspection of the funnel plot and asymmetry test to ensure the reliability of results. Sensitivity analysis and subgroup analyses were performed if necessary.

## 3 Results

### 3.1 Study selection

**Figure 1:**
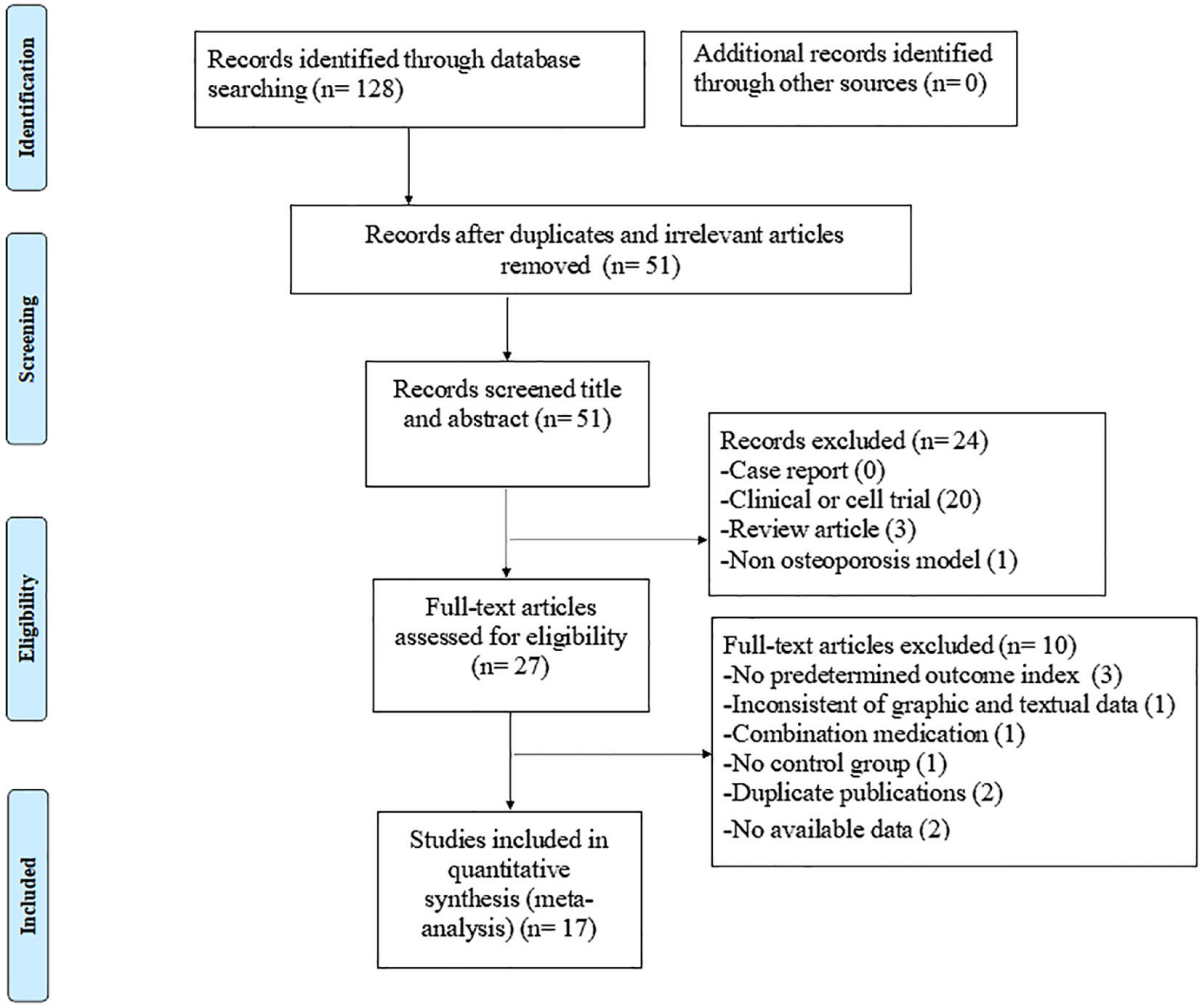
Summary of the process for identifying candidate studies.

The electronic search yielded 128 studies, of which 17 eligible studies (8, 21, 24–38) were ultimately included in this review. The specific search data and exclusion process are shown in **Figure 1**.

### 3.2 Characteristics of included studies

Seven studies published in English and 10 in Chinese, spanning from 2013 to 2024, were included. The animal models involved were female Sprague–Dawley (SD) rats (51.3%), female Wistar rats (3.9%), female C57BL/6 mice (6.4%), male SD rats (28.8%), and male ApoE^−/−^ mice (9.6%). A total of 132 rats and 25 mice were treated with Lrg; 130 rats and 25 mice served as controls. The primary outcome measures included bone pathology in three studies (26, 27, 37), F-BMD in 11 studies (21, 24, 27–31, 33–35, 39), L-BMD in three studies (8, 29, 36), Tb.Th and Tb.N in five studies (25, 28, 33, 35, 38), bone maximum load in three studies (24, 28, 30), CTX in five studies (21, 26, 31, 32, 37), OC in five studies (21, 31–34), and PINP in four studies (8, 30, 32, 37). Relevant mechanism indicators such as superoxide dismutase (SOD), tumor necrosis factor-α (TNF-α), and other detailed characteristics of the eligible studies are shown in **Table 1**.

**Table 1:** Characteristics of the included studies.

Study (years)	Species (sex, *n* = experimental/control group, weight)	Model (method)	Anesthetic	Treatment group (method to astragal sides)	Control group	Outcome index (time)
Lin 2022 (24)	Female SD rats (14/14, NM, NM)	1. Bilateral oophorectomy	Pentobarbital	By subcutaneous injection of liraglutide with 0.6 mg/kg/day after modeling and lasted 12 weeks	By subcutaneous injection of an equal volume of NS after modeling and lasted 12 weeks	1. BMD (femur) 2. Maximum load and elastic modulus 3. Serum levels of OPG and tartrate resistant acid phosphatase 4. Bone levels of FoxO3a mRNA, Wnt2 mRNA, and β⁃ndA,aat mRNA
Chong 2021 (28)	Female SD rats (12/12, 220 g, 6 to 7 weeks old)	1. Bilateral oophorectomy 2. Intraperitoneal injection of STZ (30 mg/kg) 3. High-fat and high-sugar diet	Diethyl ether	By subcutaneous injection of liraglutide with 0.1 mg/kg/day for 4 weeks after modeling; then, the daily dose was increased to 0.2 mg/kg/day for 8 weeks	By subcutaneous injection of an equal volume of NS after modeling and lasted 12 weeks	1. BMD (femur) 2. Bone-related parameters under micro-CT (Tb.N, Tb.Th, and BV/TV) 3. Maximum load, yield load, and elastic modulus 4. Content of bone mineral salt 5. Serum levels of ROS, CAT, GSH-Px, and MDA 6. Serum level of cAMP 7. Bone levels of p-PKA/PKA and p-CREB/CREB
Chen 2021 (8)	Female SD rats (10/10, 249.8 ± 56.2 g, 6 months old)	1. Bilateral oophorectomy 2. Injection of STZ (60 mg/kg)	Phenobarbital	By subcutaneous injection of liraglutide with 0.6 mg/kg/day after modeling and lasted 8 weeks	By subcutaneous injection of an equal volume of NS after modeling and lasted 8 weeks	1. BMD (lumbar) 2. Serum level of type I PINP and AKP 3. Serum level of TNF-α, IL-6, and IL-1β 4. Bone level of phosphorus and calcium
Wang 2021 (26)	Female SD rats (8/8, NM, 8 weeks old)	1. Bilateral oophorectomy 2. Intraperitoneal injection of STZ (60 mg/kg)	Chloral hydrate	By subcutaneous injection of liraglutide with 0.6 mg/kg/day after modeling and lasted 8 weeks	By subcutaneous injection of an equal volume of NS after modeling and lasted 8 weeks	1. Bone pathology 2. Serum level of ALP and CTX-1 3. TRAP activity 4. Bone level of OPG, RANKL, Runk2, and BMP 5. Bone level of STAT3 and p-STAT3
Zhang 2021 (27)	Female SD rats (10/10, 200 ± 10 g, NM)	1. Bilateral oophorectomy 2. Intraperitoneal injection of STZ (30 mg/kg) 3. High-fat and high-sugar diet	NM	By subcutaneous injection of liraglutide with 0.6 mg/kg/day after modeling and lasted 8 weeks	By subcutaneous injection of an equal volume of NS after modeling and lasted 8 weeks	1. Bone pathology 2. BMD (femur) 3. Serum level of OPG and RANKL 4. Bone level of p-PI3K, PI3K, p-Akt, and Akt
Yang 2020 (39)	Female SD rats (10/10, 220 ± 10 g, 8 weeks old)	1. Intramuscular injection of 0.1 ml dexamethasone (1 mg/kg) solution twice a week	Chloral hydrate	By subcutaneous injection of liraglutide with 0.2 mg/kg/day after modeling and lasted 12 weeks	By subcutaneous injection of an equal volume of NS after modeling and lasted 12 weeks	1. BMD (femur) 2. Bone-related parameters under Micro-CT (Tb.N and Tb.Th, BV/TV) 3. Bone level of ROS, SOD and MDA 4. Bone level of Beclin-1, At95, Map1-LC3-II, and p62/SQSTMl
Wang 2020 (29)	Female SD rats (10/10, 162.6 ± 7.4 g, 4 to 6 weeks old)	1. Bilateral oophorectomy 2. Intraperitoneal injection of STZ (35 mg/kg) 3. High-fat and high-sugar diet	Diethyl ether	By subcutaneous injection of liraglutide with 0.1 mg/kg/day for 4 weeks after modeling; then, the daily dose was increased to 0.2 mg/kg/day for 12 weeks	By subcutaneous injection of an equal volume of NS after modeling and lasted 16 weeks	1. BMD (whole body, thoracolumbar spine, bilateral femoral pelvis, and lumbar spine) 2. Blood glucose and serum insulin levels 3. Serum level of miRNA-19a and miRNA-144 4. Serum level of Cad-11/GAPDH and IRS-1//GAPDH
Subhashis 2019 (30)	Female SD rats (10/10, 250–300 g, adult)	1. Bilateral oophorectomy	Xylazine (10 mg/kg) and ketamine (40 mg/kg)	By subcutaneous injection of liraglutide with 0.6 mg/kg/day after modeling and lasted 12 weeks	By subcutaneous injection of an equal volume of water after modeling and lasted 12 weeks	1. BMD (femur) 2. Bone-related parameters under micro-CT (BV/TV and BMC) 3. Serum level of ALP, type I PINP 4. Peak load 5. Bone level of AMPK, PGC1α, and AdipoR1
Tang 2019 (31)	Male SD rats (10/10, 200–230 g, 6 to 8 weeks old)	1. Intraperitoneal injection of STZ (30 mg/kg) 2. High-fat and high-sugar diet	Chloral hydrate	By subcutaneous injection of liraglutide with 0.6 mg/kg/day after modeling and lasted 8 weeks	By subcutaneous injection of an equal volume of NS after modeling and lasted 8 weeks	1. BMD (femur) 2. Serum level of ALP, OC, OPG, RANKL, TRACR and CTX-1 3. Bone level of calcium, phosphorus, Wnt3a, LRP-5, and β-catenin
Yang 2019 (33)	Male SD rats (8/10, 220 ± 10 g, 6 to 8 weeks old)	1. Intramuscular injection of 0.1 ml dexamethasone (1 mg/kg) solution twice a week for 3 months	NM	By subcutaneous injection of liraglutide with 0.2mg/kg/day after modeling and lasted 3 months	By subcutaneous injection of an equal volume of NS after modeling and lasted 3 months	1. BMD (femur) 2. Bone-related parameters under Micro-CT (Tb.N and Tb.Th, BV/TV) 3. Serum level of TRACP, CTX-I, ALP, and OC 4. Blood glucose
Zhang 2019 (32)	Male ApoE^−/−^ mice (15/15, NM, NM)	ApoE^−/−^ mice	NM	By subcutaneous injection of liraglutide with 0.4 mg/kg/day after modeling and lasted 10 weeks	By subcutaneous injection of an equal volume of NS after modeling and lasted 10 weeks	1. Serum level of OC, PINP, PTH, CTX, and TRACP 2. Serum level of AGE, TC, and TG. 3. Bone level of RAGE-mRNA and RAGE protein
Wen 2018 (21)	Female SD rats (6/6, NM, 9 weeks old)	1. Bilateral oophorectomy 2. Intraperitoneal injection of STZ (60 mg/kg)	Chloral hydrate	By subcutaneous injection of liraglutide with 0.6 mg/kg/day after modeling and lasted 8 weeks	By subcutaneous injection of an equal volume of NS after modeling and lasted 8 weeks	1. BMD (femur) 2. Serum level of OC, OPG, CTX-I, and RANKL 3. Bone level of OPG and RANKL mRNAs
Hou 2017 (34)	Male SD rats (10/10, 338.64 ± 10.49 g, 24 weeks old)	1. High-fat diet	Chloral hydrate	By subcutaneous injection of liraglutide with 0.4 mg/kg/day after modeling and lasted 4 weeks	By subcutaneous injection of an equal volume of NS after modeling and lasted 4 weeks	1. BMD (femur) 2. Serum level of calcium and phosphorus 3. Serum level of BAP, OC, OPG, and RANKL 4. Serum level of TNF-α and IL-6 5. Bone level of OPG mRNAs and RANKL mRNAs
Huang 2016 (37)	Male SD rats (9/9, 200 ± 24 g, 6 to 8 weeks old)	1. Intraperitoneal injection of STZ (35 mg/kg) 2. High-fat and high-sugar diet	NM	By subcutaneous injection of liraglutide with 0.8 mg/kg/day after modeling and lasted 4 weeks	By subcutaneous injection of an equal volume of NS after modeling and lasted 4 weeks	1. Bone pathology 2. Serum level of calcium, phosphorus, calcitonin, 25-OH-D, PTH, FGF-23, PINP, OPG, RANKL, β-CTX, BALP, and TRAP
Zhao 2016 (36)	Male SD rats (11/7, 200–224 g, NM)	1. Intraperitoneal injection of STZ (35 mg/kg) 2. High-fat and high-sugar diet	Pentobarbital sodium	By subcutaneous injection of liraglutide with 0.8 mg/kg/day after modeling and lasted 4 weeks	By subcutaneous injection of an equal volume of NS after modeling and lasted 4 weeks	1. BMD (whole body, thoracolumbar spine, bilateral femoral pelvis, and lumbar spine) 2. Serum level of calcium, phosphorus, calcitonin, ALP, ALT, TG, and TC 3. Blood glucose and fasting insulin
Lu 2015 (35)	Female Wistar rats (6/6, NM, 6 weeks old)	1. Bilateral oophorectomy	Chloral hydrate/ether	By subcutaneous injection of liraglutide with 0.6 mg/kg/day after modeling and lasted 2 months	By subcutaneous injection of an equal volume of NS after modeling and lasted 2 months	1. BMD (femur) 2. Bone-related parameters under Micro-CT (Tb.N and Tb.Th, BV/TV) 3. Body weight and blood glucose 4. Serum level of PPARγ, ALP, Col-1, and Runx2
Pereira 2015 (38)	C57Bl/6NCrl mice (10/10, NM, 12 weeks old)	1. Bilateral oophorectomy	NM	By subcutaneous injection of liraglutide with 0.3 mg/kg/day after modeling and lasted 4 weeks	By subcutaneous injection of an equal volume of NS after modeling and lasted 4 weeks	1. Bone related parameters under micro-CT (Tb.N and Tb.Th, BV/TV) 2. Serum level of calcitonin and sclerostin 3. GLP-1 receptor in bone tissue

BMD, bone mineral density; ALP, alkaline phosphatase; GSH, glutathione peroxidase; SOD, superoxide dismutase; MDA, malondialdehyde; CAT, catalase; SD rats, Sprague–Dawley rats; TNF-α, tumor necrosis factor-α; Tb.N, trabeculae linear density; Tb.Th, trabeculae thickness; BV/TV, object surface/volume ratio; OC, osteocalcin; CTX, C-terminal cross-linked telopeptide of type I collagen; TRAP, tartrate-resistant acid phosphatase; PINP, N-terminal propeptide of type I procollagen; TRACP, tartrate-resistant acid phosphatase; BMP, bone morphogenetic protein; Runx2, runt-related transcription factor 2; NS, normal saline; RANKL, receptor activator of nuclear factor-κ B ligand; STZ, streptozotocin; Cad-11, cadherin 11; IRS-1, insulin receptor substrate-1; TC, total cholesterol; TG, triglyceride; AGE, advanced glycation end product; PPARγ, peroxisome proliferator-activated receptor γ; FoxO3a, Forkhead box protein O3a; OPG, osteoprotegerin; BMC, bone mineral content; AMPK, phosphorylated AMP-dependent protein kinase; PGC1α, peroxisome proliferator-activated receptor gamma coactivator 1-alpha; AdipoR1, adiponectin receptor 1.

### 3.3 Study quality

The number of criteria met in each study ranged from 4/10 to 8/10, with an average of 5.47. The review authors’ judgments on each risk of bias item for each included study are presented in **Table 2**.

**Table 2:** Risk of bias of the included studies.

Study	A	B	C	D	E	F	G	H	I	J	Total
Lin 2022 (24)	√	√				√			√	√	**5**
Chong 2021 (28)	√	√	√	√		√	√		√	√	**8**
Chen 2021 (8)	√	√	√			√	√			√	**6**
Wang 2021 (26)	√	√	√			√	√			√	**6**
Zhang 2021 (27)	√	√	√	√			√		√	√	**7**
Wang 2020 (29)	√		√			√	√		√		**5**
Yang 2020 (39)	√	√	√			√			√	√	**6**
Subhashis 2019 (30)	√	√		√		√			√	√	
Tang 2019 (31)	√	√	√			√				√	**5**
Yang 2019 (33)	√	√	√							√	**4**
Zhang 2019 (32)	√	√	√						√	√	**5**
Wen 2018 (21)	√		√			√	√		√	√	**6**
Hou 2017 (34)		√	√			√			√		**4**
Huang 2016 (37)	√		√						√	√	**4**
Zhao 2016 (36)	√	√	√			√			√	√	**5**
Lu 2015 (35)	√	√	√	√		√			√	√	**7**
Pereira 2015 (38)	√	√	√							√	**4**

"√" Means meeting the single criteria. Studies fulfilling the criteria of A—peer reviewed publication, B—control of temperature, C—random allocation to treatment or control, D—blinded induction of model (group randomly after modeling), E—blinded assessment of outcome, F—use of anesthetic without significant protective and toxic effects on bones, G—appropriate animal model (aged, hyperlipemia, hypertensive, or diabetes), H—sample size calculation, I—compliance with animal welfare regulations (including three or more of the following points: preoperative anesthesia, postoperative analgesia, nutrition, disinfection, environment temperature, environment humidity, circadian rhythm, and euthanasia), and J—statement of potential conflict of interests.

### 3.4 Effectiveness

#### 3.4.1 Bone pathology

Bone pathology was used as the primary outcome measure in three studies (26, 27, 37). Wang et al. (26) reported that diabetes osteoporosis rats treated with Lrg showed a marked improvement of osteoblasts on the surface of the femoral head, flattening of osteocytes, empty bone lacunae, and pyknosis of bone nuclei in the subchondral region. Two studies (27, 37) found that Lrg could increase trabecular bone and reduce trabecular bone spacing in diabetes osteoporosis rats compared with the control group.

#### 3.4.2 Bone-related parameters under imageology and bone maximum load

**Figure 2:**
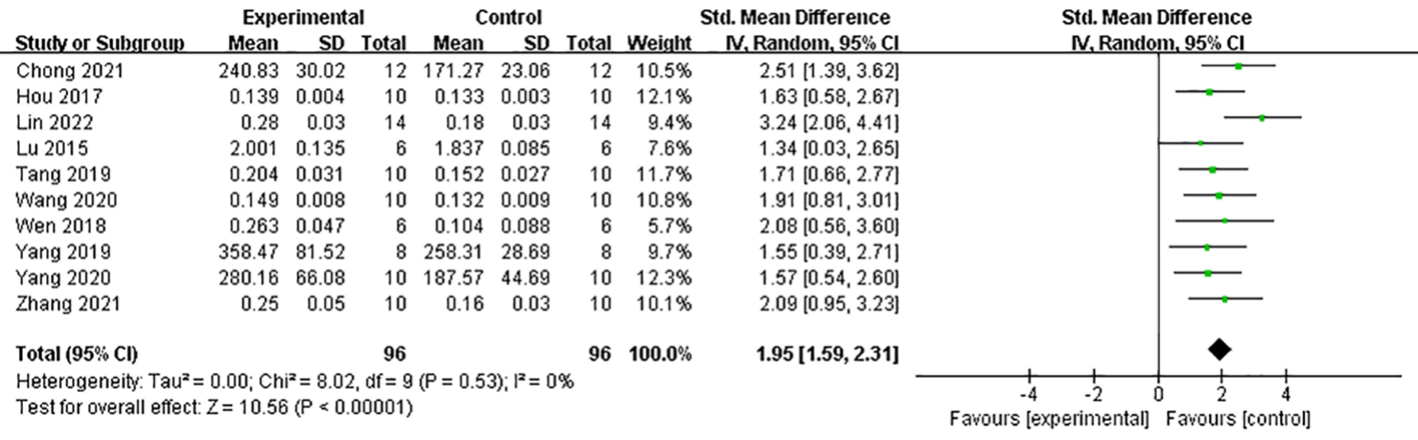
Forest plot: effects of liraglutide for increasing femur bone mineral density (F-BMD) compared with the control group.

**Figure 3:**
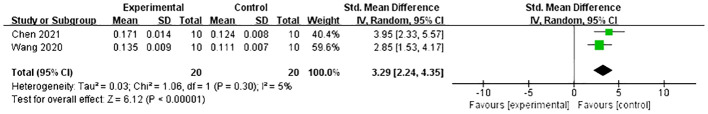
Forest plot: effects of liraglutide for increasing lumbar spine bone mineral density (L-BMD) compared with the control group.

**Figure 4:**
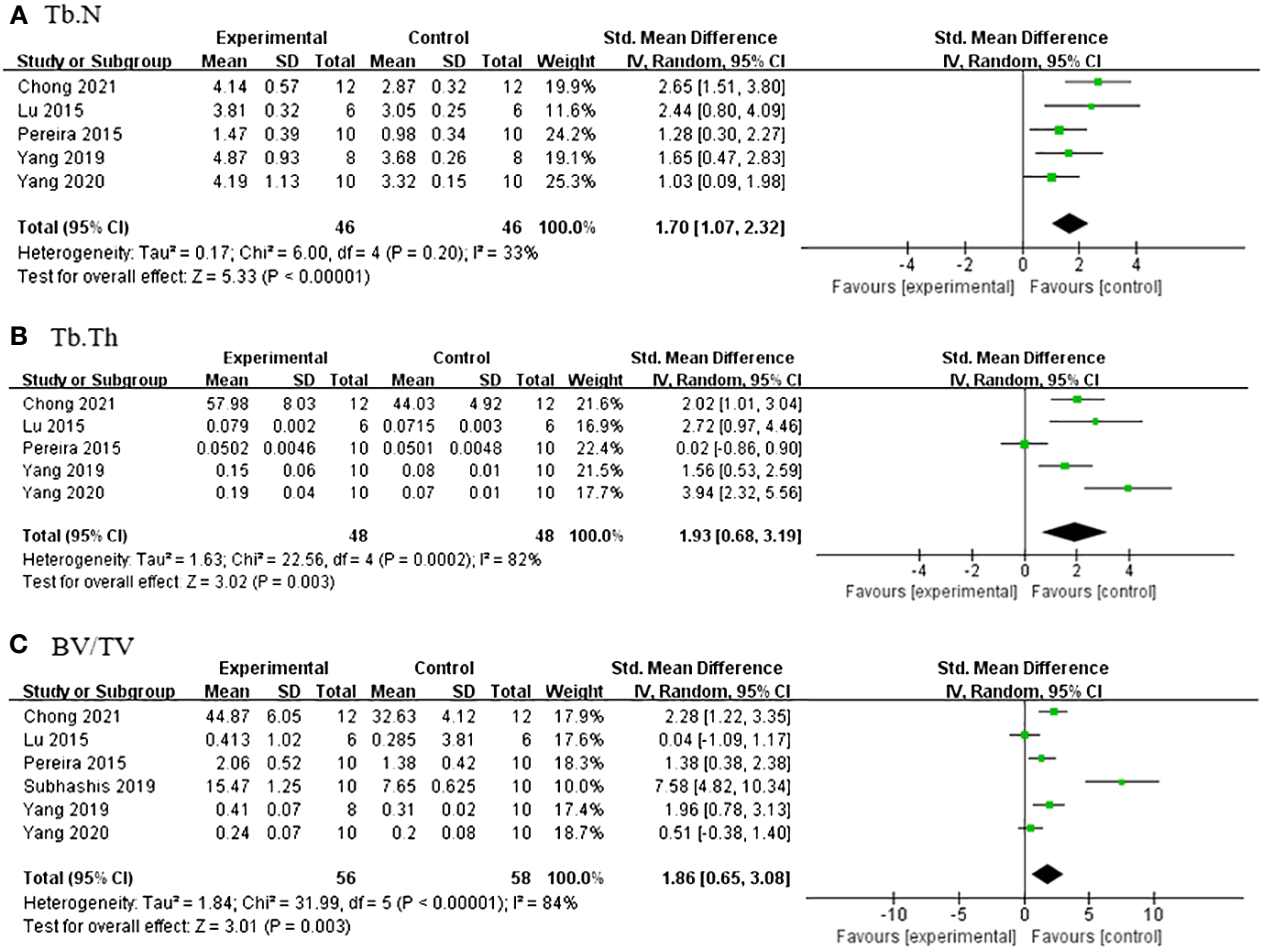
**(A)** Forest plot: effects of liraglutide for increasing trabeculae linear density (Tb.N) compared with the control group. **(B)** Forest plot: effects of liraglutide for increasing trabeculae thickness (Tb.Th) compared with the control group. **(C)** Forest plot: effects of liraglutide for increasing object surface/volume ratio (BV/TV) compared with the control group.

Under dual energy X-ray absorptiometry, 11 studies (21, 24, 27–31, 33–35, 39) reported the effect of Lrg on F-BMD. After excluding one study where the author did not specify the number of rats in each group (30), a meta-analysis of 10 studies showed a significant effect of Lrg in increasing F-BMD [*n* = 192, SMD 1.95, 95% CI (1.59, 2.31), *P* < 0.00001; heterogeneity: *χ* ^2^ = 8.02, *I* ^2^ = 0%; **Figure 2**]. Three studies (8, 29, 36) demonstrated the positive effect of Lrg on increasing L-BMD with high heterogeneity [*n* = 58, SMD 2.27, 95% CI (-0.03, 4.58), *P* < 0.00001; heterogeneity: *χ* ^2^ = 19.59, *I* ^2^ = 90%]. Sensitivity analyses of L-BMD were conducted; a meta-analysis of two studies (8, 29) showed a significant effect of Lrg in increasing L-BMD [*n* = 40, SMD 3.29, 95% CI (2.26, 4.31), *P* < 0.00001; heterogeneity: *χ* ^2^ = 1.06, *I* ^2^ = 5%; **Figure 3**] after excluding one study (36) due to differing modeling methods. Under micro-CT, a meta-analysis of five studies (25, 28, 33, 35, 38) showed significant effects of Lrg in increasing Tb.N [*n* = 92, SMD 1.65, 95% CI (1.15, 2.15), *P* < 0.00001; heterogeneity: *χ* ^2^ = 6.00, *I* ^2^ = 33%; **Figure 4A**], Tb.Th [*n* = 92, SMD 1.93, 95% CI (0.68, 3.19), *P* = 0.003; Tau^2^ = 1.63, *χ* ^2^ = 22.56, *I* ^2^ = 82%; **Figure 4B**], and BV/TV [*n* = 114, SMD 1.86, 95% CI (0.65, 3.08), *P* < 0.00001; Tau^2^ = 1.84, *χ* ^2^ = 31.99, *I* ^2^ = 84%; **Figure 4C**). Sensitivity analyses of Tb.Th and BV/TV were conducted, showing that heterogeneity did not change substantially after removing any one study. Three studies reported that Lrg could improve bone maximum load (24, 28, 30), three-point bending stress (24, 28), and elastic modulus (24, 28) (*P* < 0.05) compared with the control group.

#### 3.4.3 Serum OC, PINP, and CTX

**Figure 5:**
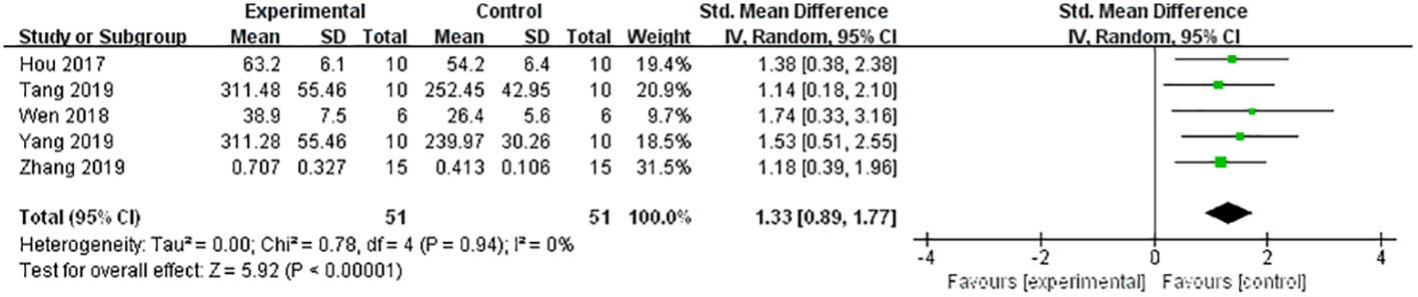
Forest plot: effects of liraglutide for increasing the level of osteocalcin (OC) compared with the control group.

A meta-analysis of five studies (21, 31–34) demonstrated a significant effect of Lrg in increasing OC [*n* = 102, SMD 1.33, 95% CI (0.89, 1.77), *P* < 0.00001; heterogeneity: *χ* ^2^ = 0.78, *I* ^2^ = 0%; **Figure 5**]. Three studies (8, 30, 37) showed a significant effect of Lrg on increasing PINP (*P* < 0.05), although one study reported contrary efficacy (32) (*P* < 0.05). Additionally, five studies (21, 26, 31, 32, 37) reported that Lrg could reduce serum CTX compared with the control group (*P* < 0.05).

#### 3.4.4 Subgroup analysis

**Figure 6:**
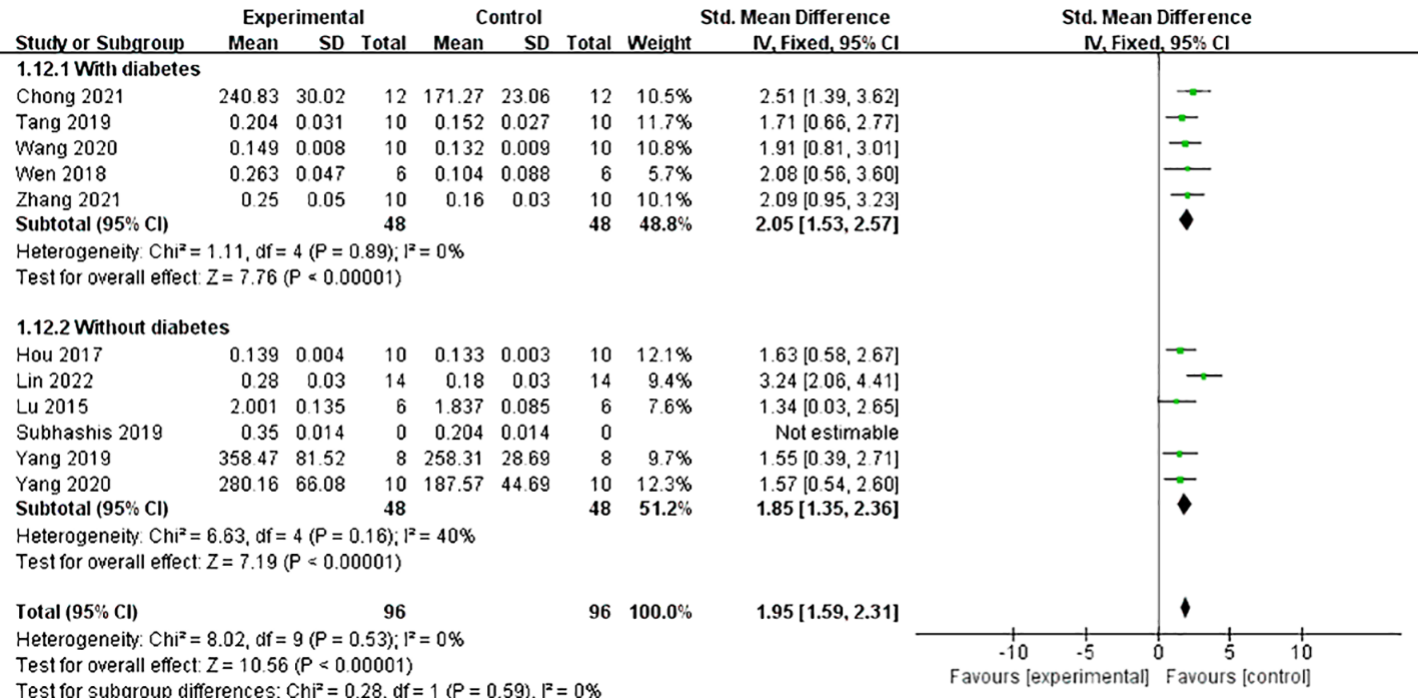
Forest plot: effects of liraglutide on femur bone mineral density in the subgroup of whether diabetes is combined.

Does the combination of diabetes make a difference in the effect of Lrg on bone resorption? We conducted a subgroup analysis on the primary outcome measure BMD, considering whether diabetes was present. The results indicated that, although the difference was not statistically significant, the effect value of Lrg in the osteoporosis with diabetes group was better than that in the osteoporosis without diabetes group (SMD 2.05 ± 0.52 vs. SMD 1.85 ± 0.50, *P* = 0.59; **Figure 6**). Although not pronounced, Lrg appears to potentially increase the efficacy by mitigating the harmful effects of high blood sugar on osteoporosis while combating the disease itself. Further animal research is required to verify this potential advantage in the future.

#### 3.4.5 Mechanism indicators

**Figure 7:**
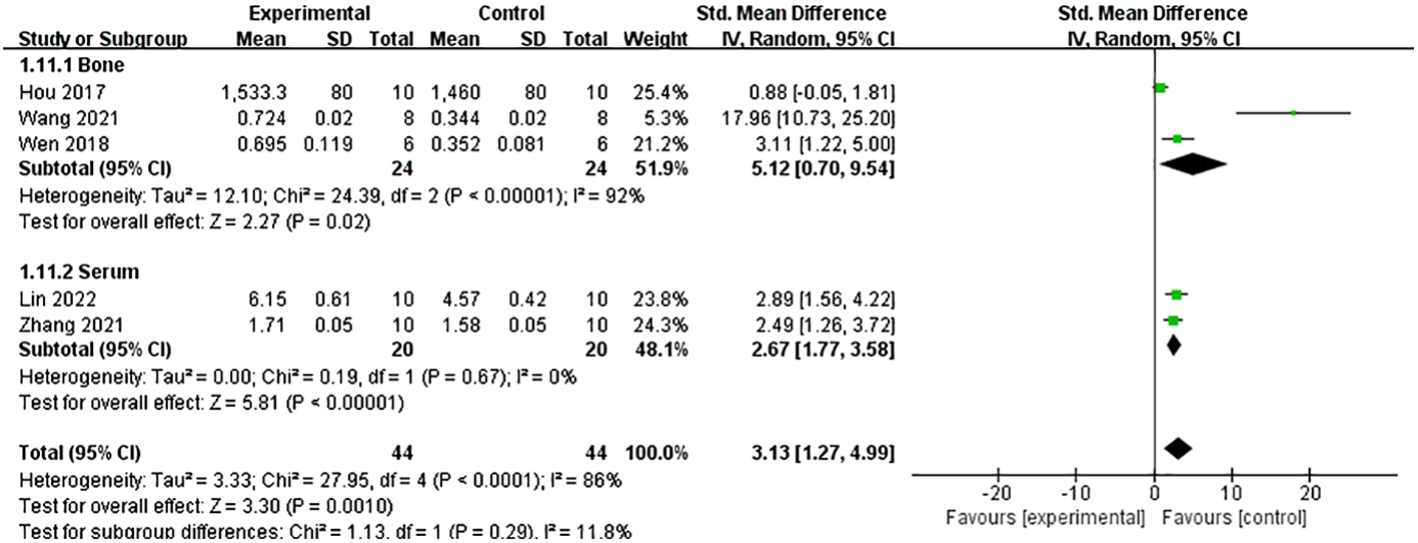
Forest plot: effects of liraglutide for increasing the level of osteoprotegerin (OPG) both in serum and bone compared with the control group.

**Figure 8:**
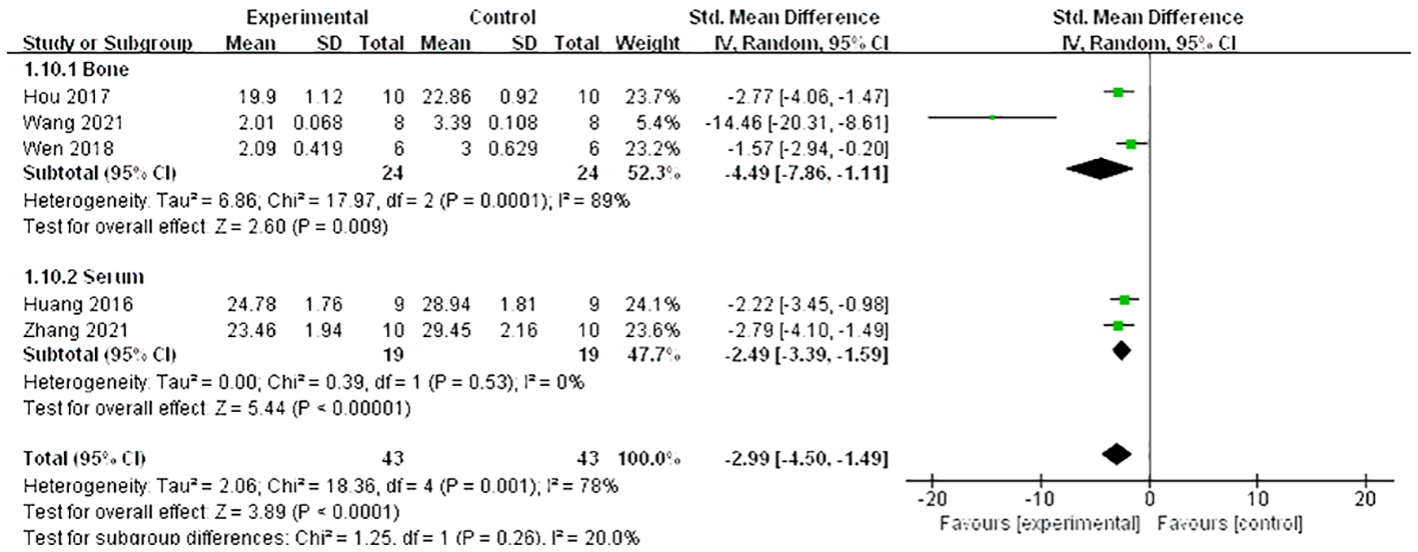
Forest plot: effects of liraglutide for reducing the level of receptor activator of nuclear factor-κ B ligand (RANKL) both in serum and bone compared with the control group.

A meta-analysis of five studies (21, 26, 27, 34, 37) demonstrated that Lrg could increase the level of osteoprotegerin (OPG) both in serum and bone [*n* = 88, SMD 3.13, 95% CI (1.27, 4.99), *P* = 0.001; heterogeneity: Tau^2^ = 3.33, *χ* ^2^ = 27.95, *I* ^2^ = 86%; **Figure 7**], and the heterogeneity of serum OPG was low (Tau² = 0.00; *χ* ^2^ = 0.19, *I*² = 0; **Figure 7**). A meta-analysis of five studies (21, 24, 26, 27, 34) also showed that Lrg could reduce the level of receptor activator of nuclear factor-kappa B ligand (RANKL) both in serum and bone [*n* = 86, SMD 2.99, 95% CI (1.49, 4.50), *P* < 0.0001; heterogeneity: Tau^2^ = 2.06, *χ* ^2^ = 18.36, *I* ^2^ = 78%; **Figure 8**], and the heterogeneity of serum RANKL was low (Tau² = 0.00; *χ* ^2^ = 0.39, *I*² = 0; **Figure 8**). STAT3 was reported as a potential target activated by Lrg to downregulate RANKL/OPG (*P* < 0.05) (26). Two studies (8, 34) indicated that Lrg could significantly reduce the levels of TNF-α, interleukin-6, and interleukin-1β (*P* < 0.05). Another two studies (25, 28) reported significant reductions in the level of reactive oxygen species (ROS) and malondialdehyde (MDA) (*P* < 0.05). Chong et al. also reported that the levels of superoxide dismutase (SOD), catalase (CAT), and glutathione peroxidase (GSH) were increased by Lrg (*P* < 0.05) (28). Lrg was found to significantly reduce the levels of Beclin-1, Atg5, and Map1-LC3-II and increase the level of p62/SQSTMl (25). Furthermore, some studies reported that Lrg could increase the levels of Wnt3a, low-density lipoprotein receptor-related protein 5 (LRP-5), β-catenin (31), adiponectin receptor 1 (AdipoR1), and peroxisome proliferator-activated receptor gamma coactivator 1-alpha (PGC1α) in osteoblasts (30).

## 4 Discussion

### 4.1 Summary of evidence

This is the first animal systematic review to include 17 studies with acceptable quality that estimate the efficacy and mechanisms of Lrg in models of osteoporosis. The findings indicate that Lrg possesses anti-osteoporosis potential while also lowering blood glucose levels.

### 4.2 Limitations

Several limitations exist within the current studies: (1) the potential for negative studies to exaggerate the efficacy of Lrg in osteoporosis due to reporting biases, (2) selection bias is likely due to the exclusive search of Chinese and English language databases, (3) methodological deficiencies are evident in the lack of blinded induction of models, blinded assessment of outcomes, and adequate sample size calculations, (4) the impact of obesity factors on osteoporosis remains controversial (40)—thus, caution is advised in interpreting results from studies treating osteoporosis caused by hyperlipidemia with liraglutide, (5) no study reported on disinfection during invasive procedures such as intraperitoneal injections, subcutaneous injections, and blood glucose measurements, which are crucial in maintaining integrity in animal studies, especially diabetic models, (6) the majority of studies did not document the incidence of rats being dropped due to complications during the modeling process, and (7) few studies addressed bone pathology directly.

### 4.3 Implication

In terms of methodology, although most studies met the scoring points of the CAMARADES 10-item quality checklist (23), the absence of crucial standards such as blinded induction of models, blinded assessment of outcomes, and rigorous sample size calculations could undermine the reliability of the findings. Adherence to the ARRIVE guidelines (41) is recommended to address these issues. When describing sample size calculation, the rational for the number of animals used should be clearly stated along with details of any calculations performed (42, 43). Measures taken to minimize the effects of subjective bias when allocating animals to treatments (e.g., randomization procedures) and when assessing results (e.g., details on who was blinded and at what stage) should also be documented. Wang et al. (44) provided a robust example of how to describe sample size, random grouping post-modeling, and blinded evaluation of outcomes.

Experimental animals with comorbidities such as advanced age, obesity, hypertension, hyperglycemia, or other risk factors may more closely mirror the physiology of patients with osteoporosis, potentially increasing the clinical relevance of research findings (45). However, it is necessary to adjust modeling approaches, such as drug dosage and mode of administration to optimize the success rate and safety of complex models in animals. In the included studies, six utilized an ovariectomized osteoporosis model with diabetes (8, 21, 26–29). Based on bilateral oophorectomy, three studies (8, 21, 26) established a diabetes model using an intraperitoneal injection of STZ at doses greater than or equal to 60 mg/kg; three studies (27–29) used STZ doses between 30 and 35 mg/kg combined with a high-fat and high-sugar diet. The inappropriate use of high doses of STZ has been associated with increased animal suffering and mortality (46). Previous studies (46–48) have shown that doses of 60 mg/kg body weight and above can be harmful or lethal to rats. Therefore, it is inappropriate to inject large doses of STZ in conjunction with major surgical models such as bilateral ovariectomy without concurrently reporting mortality, side effects, and corresponding treatments (8, 21, 26). The multiple dosage usage of STZ in the composite model is detailed in **Table 3** (49–58). The authors recommend that low-dose STZ or low-dose STZ plus high-fat feeding may be more suitable for composite models. Moreover, it is worth noting that almost all included diabetes models used intraperitoneal instead of intravenous injections of STZ for modeling. This is significant as an accidental delivery of STZ into the sub-peritoneal or bowel space may decrease the success rate and increase the mortality (46, 59). Osteoporosis models with increased bone resorption as the dominant mechanism, including ovariectomized osteoporosis, diabetic osteoporosis, and glucocorticoid models, were used in the present studies. It is suggested that future composite models can be based on other osteoporosis models rather than solely on the ovariectomized osteoporosis model.

**Table 3:** Discrepancies between blood glucose levels, type of diabetes, and mortality with varying doses of STZ.

Dose of STZ and route of administration	Efficiency (blood glucose level)	Type of diabetes	Animal	Comments	Mortality	References
70 mg/kg (single i.p./i.v.)	500 mg/dL	Type 1	Wistar rats	Lethal end point	100%	(40)
65 mg/kg (single i.p./i.v.)	350–500 mg/dL	Type 1	Albino rats	Gastric ulcerations, decrease in muscle mass and bone volume, reproductive dysfunction, nephrotoxicity, and bronchial exacerbations	20–50%	(41, 42)
55 mg/kg (single i.p./i.v.)	450 mg/dL	Type 1	Albino rats	Increased LVEDP, decreased body weight, and nephrotoxicity	10–30%	(43)
45 mg/kg (single i.p./i.v.)	300–400 mg/dL	Type 1	Wistar rats	Cardiovascular complications, decreased body weight	10%	(44, 45)
40 mg/kg (qd for 5d, i.p.)	300 mg/dL	Type 2	SD rats	Stable hyperglycemia, significantly higher kidney weight, kidney/body weight ratio, and greater impairment of kidney function	0	(46)
High-fat diet + 35 mg/kg (single i.p.)	16.7 mmol/L	Type 2	SD rats	Stable hyperglycemia, low nephrotoxicity, and decreased body weight	NM/7.14%	(47, 48)
30 mg/kg (twice/day, i.p.)	250 mg/dL	Type 2	Wistar rats	Stable hyperglycemia and low nephrotoxicity	0	(49)

**Figure 9:**
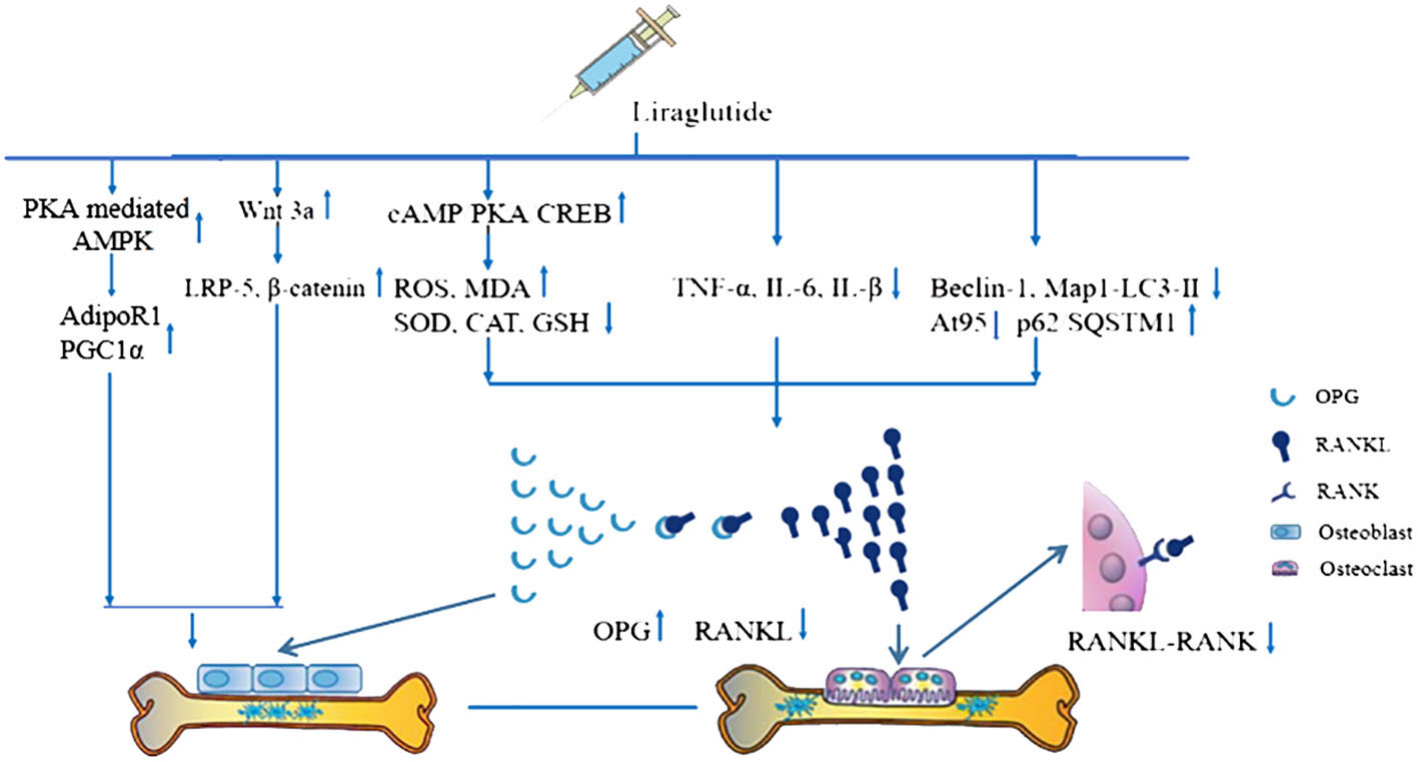
Schematic representation of osteoprotective mechanisms of liraglutide for osteoporosis.

The possible mechanisms of Lrg-mediated bone protection from the current findings are summarized as follows: (1) In bone tissue, OPG competitively binds to RANKL, blocking its blinding to RANK on the surface of osteoclasts, thus inhibiting osteoclast maturation (60). Studies indicate that the OPG/RANKL/RANK signaling pathway is increased to counteract bone resorption after Lrg treatment. STAT3 has been identified as a potential target activated by Lrg to upregulate OPG/RANKL (*P* < 0.05) (26); (2) The levels of OPG, RANKL, and RANK are regulated by a variety of cytokines and hormones that either promote or inhibit osteoclast formation, including parathyroid hormone (61), estrogen (62), 1,25(OH)2D3 (63), TNF-α, and IL-6 (64). Lrg exhibits anti-inflammatory effects by reducing the levels of TNF-α, IL-6, and IL-β (*P* < 0.05) (8, 34); (3) ROS, a critical factor in bone remodeling and homeostasis, promotes osteoclast differentiation, accelerates bone resorption, and contributes to a reduction in trabecular bone mass (65, 66). It is regulated by protective antioxidant enzymes, such as MDA, SOD, and CAT, which subsequently inhibit RANKL-induced osteoclastogenesis (67). Lrg is reported to reduce ROS and MDA levels (25, 28) and increase SOD, CAT, and GSH (28) (*P* < 0.05) through the cAMP/PKA/CREB pathway (28); (4) Excessive autophagy can lead to bone cell apoptosis or transition to autophagic death, causing bone metabolism disorders (25). Yang et al. reported that Lrg could inhibit excessive autophagy by reducing the levels of Beclin-1, Atg5, and Map1-LC3-II and increasing the level of p62/SQSTM1 under the intervention of high-dose dexamethasone (25); (5) Wnt3a, an upstream signaling molecule of the classical Wnt signaling pathway, binds with receptor LRP5 to recruit β-catenin into cells and translocate it to the nucleus, subsequently regulating bone proliferation genes and promoting osteoblast proliferation (68, 69). Lrg has been found to increase the levels of Wnt3a, LRP-5, and β-catenin (31). Moreover, activation of AdipoR1 in osteoblasts results in the upregulation of PGC1α, a mitochondrial biogenesis factor, leading to its osteogenic effect. Subhashis et al. reported that Lrg upregulated AdipoR1 and PGC1α through PKA-mediated AMPK stimulation of mitochondrial function in osteoblasts (30). The mechanism diagram is summarized in **Figure 9**.

### 4.4 Conclusion

The pre-clinical evidence reveals that Lrg is capable of partially reversing osteopenia in animal models likely by activating osteoblast proliferation through promoting the Wnt signal pathway and p-AMPK/PGC1α signal pathway and inhibiting the activation of osteoclasts by inhibiting the OPG/RANKL/RANK signal pathway through anti-inflammatory, anti-oxidant, and anti-autophagic pathways. This finding may help to improve the priority of Lrg in the treatment of diabetes patients with osteoporosis.

## Data availability statement

The original contributions presented in the study are included in the article/**Supplementary Material**. Further inquiries can be directed to the corresponding authors.

## Author contributions

ZW: Writing – original draft, Writing – review & editing. WD: Writing – original draft, Writing – review & editing. YY: Conceptualization, Data curation, Software, Writing – original draft. JX: Conceptualization, Data curation, Formal analysis, Funding acquisition, Investigation, Methodology, Project administration, Resources, Software, Supervision, Validation, Visualization, Writing – review & editing. DH: Data curation, Funding acquisition, Methodology, Resources, Supervision, Visualization, Writing – review & editing. YZ: Writing – original draft, Writing – review & editing. QZ: Conceptualization, Data curation, Formal analysis, Funding acquisition, Investigation, Methodology, Project administration, Resources, Software, Supervision, Validation, Visualization, Writing – original draft, Writing – review & editing.

## References

[1] Kanis JA Melton LJ Christiansen C Johnston CC Khaltaev N . The diagnosis of osteoporosis. J Bone Miner Res. (1994) 9:1137–41. doi: 10.1002/jbmr.5650090802 7976495

[2] Gregson CL Armstrong DJ Bowden J Cooper C Edwards J Gittoes NJL . UK clinical guideline for the prevention and treatment of osteoporosis. Arch Osteo. (2022) 17:58. doi: 10.1007/s11657-022-01061-5 PMC8979902 35378630

[3] Lane NE . Epidemiology, etiology, and diagnosis of osteoporosis. Am J Obstet Gynecol. (2006) 194:3–11. doi: 10.1016/j.ajog.2005.08.047 16448873

[4] Chinese Association of Rheumatology and Immunology Physicians Chinese Rheumatology Association Chinese Society of Bone and Mineral Research National Clinical Research Center for Dermatologic and Immunologic Diseases . Chinese consensus on the prevention and treatment of glucocorticoid induced osteoporosis. Zhong Hua Nei Ke Za Zhi. (2021) 60:13–21. doi: 10.3760/cma.j.cn112138-20201102-00914 33397016

[5] Ruiz HH Díez RL Arivazahagan L Ramasamy R Schmidt AM . Metabolism, obesity, and diabetes mellitus. Arterioscler Thromb Vasc Biol. (2019) 39:166–74. doi: 10.1161/ATVBAHA.119.312005 PMC6693645 31242034

[6] Kurra S Fink DA Siris ES . Osteoporosis-associated fracture and diabetes. Endocrinol Metab Clin North Am. (2014) 43:233–43. doi: 10.1016/j.ecl.2013.09.004 24582100

[7] Vestergaard P . Discrepancies in bone mineral density and fracture risk in patients with type 1 and type 2 diabetes-a meta-analysis. Osteo Int. (2007) 18:427–44. doi: 10.1007/s00198-006-0253-4 17068657

[8] Chen K Wu R Mo B Yan X Shen D Chen M . Comparison between liraglutide alone and liraglutide in combination with insulin on osteoporotic rats and their effect on bone mineral density. J Musculoskelet Neuro Interact. (2021) 21:142–8. PMC8020012 33657765

[9] Rizzo M Abate N Chandalia M Rizvi AA Giglio RV Nikolic D . Liraglutide reduces oxidative stress and restores heme oxygenase-1 and ghrelin levels in patients with type 2 diabetes: a prospective pilot study. J Clin Endocrinol Metab. (2015) 100:603–6. doi: 10.1210/jc.2014-2291 PMC4318909 25393640

[10] Giglio RV Nikolic D Volti GL Stoian AP Banerjee Y Magan-Fernandez A . Liraglutide increases serum levels of microRNA-27b, -130a and -210 in patients with type 2 diabetes mellitus: A novel epigenetic effect. Metabolites. (2020) 10:391. doi: 10.3390/metabo10100391 33008044 PMC7599907

[11] Patti AM Rizvi AA Giglio RV Stoian AP Ligi D Mannello F . Impact of glucose-lowering medications on cardiovascular and metabolic risk in type 2 diabetes. J Clin Med Mar. (2020) 9:912. doi: 10.3390/jcm9040912 PMC7230245 32225082

[12] Nikolic D Patti AM Giglio RV Chianetta R Castellino G Magán-Fernández A . Liraglutide improved cardiometabolic parameters more in obese than in non-obese patients with type 2 diabetes: A real-world 18-month prospective study. Diabetes Ther. (2022) 13:453–64. doi: 10.1007/s13300-022-01217-z PMC8853434 35167051

[13] Rizzo M Rizvi AA Patti AM Nikolic D Giglio RV Castellino G . Liraglutide improves metabolic parameters and carotid intima-media thickness in diabetic patients with the metabolic syndrome: an 18-month prospective study. Cardiovasc Diabetol. (2016) 15:162. doi: 10.1186/s12933-016-0480-8 27912784 PMC5135832

[14] Patti AM Nikolic D Magan-Fernandez A Giglio RV Castellino G Chianetta R . Exenatide once-weekly improves metabolic parameters, endothelial dysfunction and carotid intima-media thickness in patients with type-2 diabetes: an 8-month prospective study. Diabetes Res Clin Pract. (2019) 149:163–9. doi: 10.1016/j.diabres.2019.02.006 30759365

[15] Giglio RV Pantea SA Al-Rasadi K Banach. M Patti AM Ciaccio M . Novel therapeutical approaches to managing atherosclerotic risk. Int J Mol Sci. (2021) 22:4633. doi: 10.3390/ijms22094633 33924893 PMC8125277

[16] Patti AM Giglio RV Papanas N Rizzo M Rizvi AA . Future perspectives of the pharmacological management of diabetic dyslipidemia. Expert Rev Clin Pharmacol. (2019) 12:129–43. doi: 10.1080/17512433.2019.1567328 30644763

[17] Su B Sheng H Zhang M Bu L Yang P Li L . Risk of bone fractures associated with glucagon-like peptide-1 receptor agonists’ treatment: a meta-analysis of randomized controlled trials. Endocrine. (2015) 48:107–15. doi: 10.1007/s12020-014-0361-4 25074632

[18] Iepsen EW Lundgren JR Hartmann B Pedersen O Hansen. T Jørgensen NR . GLP-1 receptor agonist treatment increases bone formation and prevents bone loss in weight-reduced obese women. J Clin Endocrinol Metab. (2015) 100:2909–17. doi: 10.1210/jc.2015-1176 26043228

[19] Rubino DM Greenway FL Khalid U O’Neil PM Rosenstock J Sørrig R . Effect of weekly subcutaneous semaglutide vs daily liraglutide on body weight in adults with overweight or obesity without diabetes: the STEP 8 randomized clinical trial. JAMA. (2022) 327:138–50. doi: 10.1001/jama.2021.23619 PMC8753508 35015037

[20] Nuche-Berenguer B Portal-Núñez S Moreno P González N Acitores A López-Herradón A . Presence of a functional receptor for GLP-1 in osteoblastic cells, independent of the cAMP-linked GLP-1 receptor. J Cell Physiol. (2010) 225:585–92. doi: 10.1002/jcp.22243 20506394

[21] Wen B Zhao L Zhao H Wang X . Liraglutide exerts a bone-protective effect in ovariectomized rats with streptozotocin-induced diabetes by inhibiting osteoclastogenesis. Exp Ther Med. (2018) 15:5077–83. doi: 10.3892/etm.2018.6043 PMC5958780 29805533

[22] Moher D Liberati A Tetzlaff J Altman DG PRISMA Group . Preferred reporting items for systematic reviews and meta-analyses: the PRISMA statement. PloS Med. (2009) 6:e1000097. doi: 10.1371/journal.pmed.1000097 19621072 PMC2707599

[23] Macleod MR O’Collins T Howells DW Donnan GA . Pooling of animal experimental data reveals influence of study design and publication bias. Stroke. (2004) 35:1203–8. doi: 10.1161/01.STR.0000125719.25853.20 15060322

[24] Lin X Sun QY Xu JL . Liraglutide improves bone mineral density in osteoporotic rats by mediating O3a/Wnt signaling. Chin J Endocr Surg. (2022) 16:221–5. doi: 10.3760/cma.j.cn.115807-20211215-00388

[25] Yang C Tao H Zhang H Xia Y Bai J Ge G . TET2 regulates osteoclastogenesis by modulating autophagy in OVX-induced bone loss. Autophagy. (2022) 18:2817–29. doi: 10.1080/15548627.2022.2048432 PMC9673923 35255774

[26] Wang X Mi Y He W Yang S Zhao L Zhang Y . Effect of liraglutide on regulation of bone metabolism in diabetic osteoporotic rats by activating STAT3. Chin J Osteo Bone Miner Res. (2021) 14:495–503. doi: 10.3969/j.issn.1674-2591.2021.05.007

[27] Zhang Y Zhou YH Li JY Zhao JL Wu SY Xiong CY . Effect of liraglutide on type 2 diabetic osteoporosis rats based on the study of PI3K/Akt pathway. Chin J Osteo. (2021) 27:985–9. doi: 10.3969/j.issn.1006-7108.2021.07.009

[28] Chong XJ Yang LX . Mechanism of liraglutide intervening osteoporosis in type 2 diabetic rats through c AMP/PKA/CREB signaling pathways. Chin J Pathophysiol. (2021) 37:1949–56. doi: 10.3969/j.issn.1000-4718.2021.11.004

[29] Wang Z Liu J . Effects of liraglutide on microRNA-19a, microRNA-144, and bOne mineral density in osteoporotic rats with type 2 diabetes mellitus. Chin J Osteo. (2020) 26:1297–300. doi: 10.3969/j

[30] Subhashis P Shailendra KM Sourav C Shyamsundar PC Konica P Chirag K . The osteogenic effect of liraglutide involves enhanced mitochondrial biogenesis in osteoblasts. Biochem Pharmacol. (2019) 164:34–44. doi: 10.1016/j.bcp.2019.03.024 30885766 PMC7234838

[31] Tang XB Pan CY Lou Y . Effects of liraglutide on bone metabolism and Wnt pathway in type 2 diabetic rats with osteoporosis. J Endocr Surge. (2019) 13:466–70. doi: 10.3760/cma.j.issn.1674-6090.2019.06.006

[32] Zhang L Li P Tang Z Dou Q Feng B . Effects of GLP-1 receptor analogue liraglutide and DPP-4 inhibitor vildagliptin on the bone metabolism in ApoE-/-mice. Ann Transl Med. (2019) 7:369. doi: 10.21037/atm.2019.06.74 31555683 PMC6736830

[33] Yang L Yang J Pan T Zhong X . Liraglutide increases bone formation and inhibits bone resorption in rats with glucocorticoid-induced osteoporosis. J Endocrinol Invest. (2019) 42:1125–31. doi: 10.1007/s40618-019-01034-5 30955181

[34] Hou DL . Study on effects of Liraglutide on bone mineral density and bone brittle degree in rats of high-fat diet inducing obesity and the underlying mechanism. Hebei Med Univ. (2017).

[35] Lu N Sun H Yu J Wang J Liu M Zhao L . Glucagon-like peptide-1 receptor agonist Liraglutide has anabolic bone effects in ovariectomized rats without diabetes. PloS One. (2015) 10:132744. doi: 10.1371/journal.pone.0132744 PMC4503456 26177280

[36] Zhao Y Wang Y Zhang Y Song DP Wang QP . The effect of GLP-1 on serum concentrations of OPG and RANKL and bone mineral density in type 2 diabetic rats. Chin J Osteo. (2016) 22:700–5. doi: 10.3969/j.issn.1006-7108.2016.06.009

[37] Huang SY Fu JY Li H Zhao Y Li Y Yang QP . Effects of liraglutide on bone metabolism and bone microstructure in type 2 diabetic rats. Chin J diab. (2016) 24:1111–5. doi: 10.3969/j.issn.1006-6187.2016.12.012

[38] Pereira M Jeyabalan J Jørgensen CS Hopkinson M Al-Jazzar A Roux JP . Chronic administration of Glucagon-like peptide-1 receptor agonists improves trabecular bone mass and architecture in ovariectomised mice. Bone. (2015) 81:459–67. doi: 10.1016/j.bone.2015.08.006 26314515

[39] Yang J Yang LN Ding TT Zhong X Pan TR . Liraglutide relieves glucocorticoid osteoporosis in rats through autophagy and improvement of oxidative stress. J Anhui Med University. (2020) 55:1724–8. doi: 10.19405/j.cnki.issn1000-1492.2020.11.016

[40] Rinonapoli G Pace V Ruggiero C Ceccarini P Bisaccia M Meccariello L . Obesity and Bone: A complex relationship. Int J Mol Sci. (2021) 22:13662. doi: 10.3390/ijms222413662 34948466 PMC8706946

[41] Kilkenny C Browne WJ Cuthill IC Emerson M Altman DG . Improving bioscience research reporting: the ARRIVE guidelines for reporting animal research. PLoS Biol. (2010) 8:e1000412. doi: 10.1371/journal.pbio.1000412 20613859 PMC2893951

[42] Pan YS Jin AM Wang MX . Methods and common pitfalls of sample size estimation in clinical studies. Chin J Stroke. (2022) 17:31–5. doi: 10.3969/j.issn.1673-5765.2022.01.004

[43] Hayes RJ Bennett S . Simple sample size calculation for cluster-randomized trials. Int J Epidemiol. (1999) 28:319–26. doi: 10.1093/ije/28.2.319 10342698

[44] Wang P Li Y Yan B Yang Z Li L Cao Z . Manganese porphyrin promotes post cardiac arrest recovery in mice and rats. Biology. (2022) 11:957. doi: 10.3390/biology11070957 36101338 PMC9312251

[45] Brosius FC Alpers CE Bottinger EP Breyer MD Coffman TM Gurley SB . Mouse models of diabetic nephropathy. J Am Soc Nephrol. (2009) 20:2503–12. doi: 10.1681/ASN.2009070721 PMC4075053 19729434

[46] Goyal SN Reddy NM Patil KR Nakhate KT Ojha S Patil CR . Challenges and issues with streptozotocin-induced diabetes - A clinically relevant animal model to understand the diabetes pathogenesis and evaluate therapeutics. Chem Biol Interact. (2016) 244:49–63. doi: 10.1016/j.cbi.2015.11.032 26656244

[47] Sennoune S Gerbi A Duran MJ Grillasca JP Compe E Pierre S . Effect of streptozotocin-induced diabetes on rat liver Na+/K+-ATPase. Eur J Biochem. (2000) 267:2071–8. doi: 10.1046/j.1432-1327.2000.01211.x 10727947

[48] Zhuang Z Wang ZH Huang YY Zheng Q Pan XD . Protective effect and possible mechanisms of ligustrazine isolated from ligusticum wallichii on nephropathy in rats with diabetes: A preclinical systematic review and meta-analysis. J Ethnopharmacol. (2020) 252:112568. doi: 10.1016/j.jep.2020.112568 31978520

[49] GGajdosík A Gajdosíková A Stefek M Navarová J Hozová R . Streptozotocin-induced experimental diabetes in male Wistar rats. Gen Physiol Biophys. (1999) 18:54–62. 10703720

[50] Motyl K McCabe LR . Streptozotocin, type I diabetes severity and bone. Biol Proced Online. (2009) 11:296–315. doi: 10.1007/s12575-009-9000-5 19495918 PMC3055251

[51] Zafar M Naqvi S Ahmed M Kaimkhani ZA . Altered kidney morphology and enzymes in streptozotocin induced diabetic rats. Int J Morphol. (2009) 27:783–90. doi: 10.4067/S0717-95022009000300024

[52] Saleh DO Bayoumi AR El-Eraky WI El-Khatib AS . Streptozotocininduced vascular and biochemical changes in rats: effects of rosiglitazone vs. Metformin. Bull. Fac Pharm Cairo Univ. (2013) 51:131–8. doi: 10.1016/j.bfopcu.2013.03.002

[53] Raghunathan S Tank P Bhadada S Patel B . Evaluation of buspirone on streptozotocin induced type 1 diabetes and its associated complications. Biomed Res Int. (2014) 2014:948427. doi: 10.1155/2014/948427 24563867 PMC3915896

[54] Tesseromatis C Kotsiou A Parara H Vairaktaris E Tsamouri M . Morphological changes of gingiva in streptozotocin diabetic rats. Int J Dent. (2009) 2009:725628. doi: 10.1155/2009/725628 20339569 PMC2836915

[55] Wang ZS Xiong F Xie XH Chen D Pan JH Cheng L . Astragaloside IV attenuates proteinuria in streptozotocin-induced diabetic nephropathy via the inhibition of endoplasmic reticulum stress. BMC Nephrol. (2015) 31:44. doi: 10.1186/s12882-015-0031-7 PMC4387678 25886386

[56] Ju Y Su Y Chen Q Ma K Ji T Wang Z . Protective effects of Astragaloside IV on endoplasmic reticulum stress-induced renal tubular epithelial cells apoptosis in type 2 diabetic nephropathy rats. Biomed Pharmacother. (2019) 109:84–92. doi: 10.1016/j.biopha.2018.10.041 30396095

[57] Ma KK Ju YH Chen QQ Li WZ Li WP . Effect of astragaloside IV on regulation of PI3K/akt/foxO1 signal in kidney of type 2 diabetic nephropathy rats. Chin J Exp Tradit Med Formulae. (2019) 25:74–81. doi: 10.13422/j.cnki.syfjx.20190227

[58] Zhang M Lv XY Li J Xu ZG Chen L . The characterization of high-fat diet and multiple low-dose streptozotocin induced type 2 diabetes rat model. Exp Diabetes Res. (2008) 2008:704045. doi: 10.1155/2008/704045 19132099 PMC2613511

[59] Zaben KRA . Induction of diabetes mellitus in rats using intraperitoneal streptozotocin: a comparison between 2 strains of rats. Eur J Sci Res. (2009) 32:398–402.

[60] Nagy V Penninger JM . The RANKL-RANK story. Gerontology. (2015) 61:534–42. doi: 10.1159/000371845 25720990

[61] Kužma M Jackuliak P Killinger Z Payer J . Parathyroid hormone-related changes of bone structure. Physiol Res. (2021) 70:3–11. doi: 10.33549/physiolres.934779 34918524 PMC8884379

[62] Cheng CH Chen LR Chen KH . Osteoporosis due to hormone imbalance: an overview of the effects of estrogen deficiency and glucocorticoid overuse on bone turnover. Int J Mol Sci. (2022) 23:1376. doi: 10.3390/ijms23031376 35163300 PMC8836058

[63] Takahashi N Akatsu T Sasaki T Nicholson GC Moseley JM Martin TJ . Induction of calcitonin receptors by 1 alpha, 25-dihydroxyvitamin D3 in osteoclast-like multinucleated cells formed from mouse bone marrow cells. Endocrinology. (1988) 123:1504–10. doi: 10.1210/endo-123-3-1504 2841098

[64] Ghosh R Dey R Sawoo R Haque W Bishayi B . Endogenous neutralization of TGF-β and IL-6 ameliorates septic arthritis by altering RANKL/OPG interaction in lymphocytes. Mol Immunol. (2022) 152:183–206. doi: 10.1016/j.molimm.2022.10.015 36371814

[65] Xian Y Su Y Liang J Long F Feng X Xiao Y . Oroxylin a reduces osteoclast formation and bone resorption via suppressing RANKL-induced ROS and NFATc1 activation. Biochem Pharmacol. (2021) 193:114761. doi: 10.1016/j.bcp.2021.114761 34492273

[66] Sendur OF Turan Y Tastaban E Serter M . Antioxidant status in patients with osteoporosis: a controlled study. Joint Bone Spine. (2009) 76:514–8. doi: 10.1016/j.jbspin.2009.02.005 19464221

[67] Hyeon S Lee H Yang Y Jeong W . Nrf2 deficiency induces oxidative stress and promotes RANKL-induced osteoclast differentiation. Free Radical Biol Med. (2013) 65:789–99. doi: 10.1016/j.freeradbiomed.2013.08.005 23954472

[68] Holloway-Kew KL De Abreu LLF Kotowicz MA Sajjad MA Pasco JA . Bone turnover markers in men and women with impaired fasting glucose and diabetes. Calcified Tissue Int. (2019) 104:599–604. doi: 10.1007/s00223-019-00527-y 30680432

[69] Sebastian A Hum NR Murugesh DK Hatsell S Economides AN Loots GG . Wnt co-receptors Lrp5 and Lrp6 differentially mediate Wnt3a signaling in osteoblasts. PloS One. (2017) 12:188264. doi: 10.1371/journal.pone.0188264 PMC5703471 29176883

