# Supplementary material for: Liraglutide, a glucagon-like peptide-1 receptor agonist, inhibits bone loss in an animal model of osteoporosis with or without diabetes
Source: Front Endocrinol (Lausanne). 2024 May 29;15:1378291. doi: 10.3389/fendo.2024.1378291 (PMC11167098; doi:10.3389/fendo.2024.1378291)
Supplement: Supplementary file 1 [file DataSheet_1.pdf]

Pubmed search strategy

1. Pubmed	
#1	Search (Liraglutide [Title/Abstract]) OR (Victoza [Title/Abstract])
#2	Search (((Osteoporosis) OR (Bone Loss)) OR (Bone Metabolism)) OR (Osteopenia)
#3	#1 AND #2
#4	Filters: Publication date to 2024/04/27.<br>Items found: 65
